# Clinical presentation, management, and outcomes of post-transplant lymphoproliferative disorder in renal and pancreas transplantation: a 22-year experience

**DOI:** 10.3389/frtra.2026.1830197

**Published:** 2026-06-17

**Authors:** Kian Emami, Jane Norman, Titus Augustine

**Affiliations:** 1 Faculty of Biology, Medicine and Health, The University of Manchester, Manchester, United Kingdom; 2 Department of Haematology, Manchester Royal Infirmary, Manchester University NHS Foundation Trust, Manchester, United Kingdom; 3 Manchester Centre for Transplantation, Manchester Royal Infirmary, Manchester University NHS Foundation Trust, Manchester, United Kingdom

**Keywords:** clinical presentation, diagnostic delay, epstein barr virus, immunosuppression, pancreas transplantation, post-transplant lymphoproliferative disorder, renal transplantation, survival analysis

## Abstract

**Background:**

Post-transplant lymphoproliferative disorder (PTLD) is a life-threatening complication of solid organ transplantation associated with long-term immunosuppression and Epstein–Barr virus (EBV) reactivation. This study analyses the clinical presentation, diagnostic pathways, therapeutic strategies and outcomes of PTLD in renal and pancreatic transplant recipients at the Manchester Centre for Transplantation.

**Methods:**

A retrospective cohort study was carried out on a cohort of 73 patients diagnosed with PTLD between 2002 and 2025 following renal, simultaneous pancreas-kidney (SPK) or pancreas-only transplantation. Data were collected from electronic medical records and analysed descriptively. Clinical presentations were categorised into organ system involvement. Time from presentation to diagnosis and survival outcomes were assessed.

**Results:**

The median time from transplant to PTLD diagnosis was 132 months, with 56% of cases diagnosed more than 10 years post-transplant. Monomorphic PTLD, in particular diffuse large B-cell lymphoma (DLBCL), was the most common subtype. EBV-positivity was seen in all early cases and in 57% overall. Abdominal and B symptoms were the most frequent presentations. Bleeding and/or anaemia were significantly associated with diagnostic delay (*p* = 0.045), and delays over one month were associated with reduced survival (*p* = 0.061). Complete or partial remission was achieved in 75% of patients. The 5-year overall survival rate was 68% while 1-year survival was 83% and death-censored graft survival was 82%.

**Conclusion:**

In our cohort, PTLD in transplant recipients presents with diverse symptoms and can occur several years post-transplant highlighting the need for long-term vigilance. Streamlined referral pathways and increased awareness could reduce diagnostic delays. Establishing a national PTLD registry would benefit future research.

## 1 Introduction

### 1.1 Background

Post-transplant lymphoproliferative disorder (PTLD) is a rare but serious complication of organ transplantation affecting a reported 1%–20% of solid organ transplant (SOT) recipients (1). The majority of PTLD cases are driven by chronic immunosuppression and, in many cases, latent Epstein–Barr virus (EBV) infection (2, 3). PTLD is a significant cause of post-transplant morbidity and mortality with ramifications for both graft survival and patient outcomes.

PTLD differs from lymphomas in the general population in several aspects, including its distinct histopathological features, increased extranodal involvement and generally more aggressive clinical course (4, 5). Although the overall incidence of PTLD in renal transplant populations is lower (0.8%–2.5%) in comparison to other SOT recipients, such as multiorgan and intestinal transplant recipients (up to 20%) and heart transplant recipients (2%–8%), the large number of renal transplants carried out globally makes PTLD in this population a significant clinical challenge (4, 6). Early diagnosis, management and monitoring is key to obtaining favourable outcomes in this group (7, 8). Current literature is largely composed of case reports, small case series and mixed-organ cohorts, with little detail on the presentation, diagnostic modalities, management pathways and outcomes specifically in renal transplant recipients (9–11).

### 1.2 Pathophysiology

One of the most widely accepted mechanisms in PTLD pathogenesis involves unregulated proliferation of EBV-infected B cells. Up to 95% of the adult population has had exposure to the virus where primary infection in immunocompetent individuals is usually either asymptomatic, or a self-limiting illness with minimal complications. After infection, the virus remains latent lifelong in B lymphocytes where cytotoxic T cells regulate their proliferation. In the post-transplant setting, immunosuppressive medication inhibits these T cells, removing the controls over EBV-infected B cells. This leads to unchecked activation, the acquisition of oncogenic mutations and clonal expansion (2, 12). This ultimately results in the development of PTLD.

EBV-positive PTLD can therefore occur via viral reactivation or primary EBV infection through the donated organ or environmental exposure during immunosuppression (13, 14). Consequently, EBV-seronegative recipients who acquire primary EBV infection post-transplant are at increased risk of developing PTLD (15). EBV-positive PTLD often presents early, within a year of transplantation. In contrast, EBV-negative PTLD has a distinctly separate pathogenesis that is poorly understood but hypothesised to involve the cumulative effects of long-term immunosuppression, decreased immunosurveillance, antigenic stimulation and infection with oncoviruses other than EBV (16). EBV-negative PTLD tends to present later than EBV-positive disease, often 5–15 years post transplantation.

### 1.3 Epidemiology

PTLD is the second most common malignancy among SOT recipients behind skin cancer. PTLD incidence in SOT recipients displays a bimodal distribution. An initial peak occurs within 12–24 months of transplantation with a second peak seen 5–10 years post-transplantation (5, 12, 17). Earlier incidence is associated with EBV-positivity whereas late onset PTLD tends to be EBV-negative. Historically, PTLD development tends to occur early although current data shows a rising number of late-onset, EBV-negative cases (18, 19). Several risk factors are associated with PTLD development, the most significant include: EBV-seronegativity prior to transplantation, primary EBV infection post-transplantation and high cumulative levels of immunosuppression (13). Those most at risk are EBV-seronegative patients receiving grafts from EBV-positive donors (14).

### 1.4 Clinical presentation

The clinical presentation of PTLD is heterogenous and may mimic other pathological conditions. Symptoms most frequently include classical B symptoms including weight loss, pyrexia, and night sweats, or can be organ-specific and pertaining to the sites affected. Malaise, fatigue, and a mononucleosis-like state are also common manifestations (2). Lymphadenopathy is a frequent sign that can cause compressive symptoms however extranodal involvement is also common, often involving the gastrointestinal tract (GIT), lungs, central nervous system (CNS), and bone marrow (BM). In renal transplant recipients, the GIT is the most common site of extranodal involvement often presenting with bowel obstruction, abdominal pain and changes in bowel habit (4, 20, 21). In many cases the allograft itself can be involved risking dysfunction and rejection.

### 1.5 Diagnosis

Definitive diagnosis of PTLD requires histopathological analysis through biopsy of the involved tissue. Specimens are classified according to the 2017 World Health Organisation (WHO) criteria as either: monomorphic, polymorphic, classic Hodgkin or non-destructive PTLD (22). Most PTLDs are B cell in origin with only 5% displaying T cell neoplasia (23). EBV status of tissue should be determined as part of the diagnostic workup (24). Imaging using computed tomography (CT) and positron emission tomography-computed tomography (PET-CT) is employed as both initial evaluation and staging of disease. Disease staging is generally based on the Ann Arbor system which stratifies lymphoma according to the extent of nodal involvement and the presence of extranodal disease (25). Further assessments may include BM biopsy in patients with suspected marrow involvement and brain MRI and cerebrospinal fluid analysis for patients with suspected CNS involvement.

### 1.6 Management and prognosis

The objective of treatment for PTLD is twofold: providing a curative remission of the disease while also preserving graft function. This proves a challenge as vigorous treatment of PTLD can jeopardise graft survival. Given the clinicopathologic heterogeneity of PTLD, there is no unifying treatment approach. In the UK, management of PTLD is guided by the British Society of Haematology (BSH) recommendations (24). The mainstay of PTLD management is a reduction of immunosuppression (RI) (26). This usually involves a reduction of calcineurin inhibitors and withdrawal of anti-proliferative and anti-metabolite agents. Often this is the only management required to achieve complete remission, especially in patients with early and polymorphic subtypes. Nonetheless more frequently, RI is used in conjunction with either the anti-CD20 monoclonal antibody rituximab, in cases of CD20-positive B cell PTLD, or with rituximab followed by CHOP (cyclophosphamide, doxorubicin, vincristine and prednisolone) also known as RCHOP chemotherapy (27, 28).

Local therapy such as radiotherapy targeted at the sites of disease is also common. Surgical intervention is occasionally used for diagnostic purposes and to manage complications of localised disease (2). In renal transplant recipients, the major concern is balancing effective lymphoma treatment with the risk of graft rejection. This necessitates the ongoing monitoring of renal function and resulting adjustments in immunosuppressive regimens.

### 1.7 Aims

This study aims to evaluate the clinical and histological characteristics, time to definitive diagnosis, management protocols and outcomes of PTLD in renal and pancreas transplant recipients at the Manchester Royal Infirmary Centre for Transplantation. The primary aim is to analyse time from symptomatic presentation to diagnosis of PTLD, whether this varies significantly with different presentations and its effect on outcomes. Information on immunosuppression regimens, EBV status, histology and staging will also be considered.

## 2 Methods

A single-centre retrospective analysis was carried out on a cohort of 73 patients using the hospital's electronic medical records. Inclusion criteria comprised all patients diagnosed and treated for PTLD between 2002 and 2025 following renal, SPK or pancreas-only transplantation at the Manchester Royal Infirmary. Exclusion criteria involved patients who received other types of organ transplants, had insufficient or incomplete documentation precluding meaningful analysis, or could not be identified on the electronic records system. Of 79 cases identified, 2 were excluded due to non-renal/pancreas transplants, 3 were excluded due to missing or incomplete records and 1 was excluded due to an unconfirmed PTLD yielding a final cohort of 73.

As this study was conducted within the scope of a service evaluation, Health Research Authority (HRA) ethical approval was not required in accordance with the HRA guidelines.

Data collection occurred in May 2025 using the HIVE electronic record system. Clinical information was gathered from multiple sources including outpatient clinic letters, histopathology reports, referrals, discharge summaries, A&E admission sheets, multidisciplinary team (MDT) discussions and treatment summaries. These were followed up longitudinally until the most recent documentation. Data were entered manually into a Microsoft Excel spreadsheet with all data anonymised and no patient-identifiable information used in the final analysis. No patients were contacted for data collection. The following data points were captured:

**Demographics and transplant details:**

- Age at PTLD diagnosis
- Sex
- Year and type of transplantation
- Time from transplant to PTLD (in months)

**Clinical presentation and diagnosis:**

- Presenting symptoms
- Time from symptom onset to PTLD diagnosis
- Diagnostic modality

**Disease characteristics:**

- PTLD subtype (according to WHO 2008 classification: early, polymorphic, monomorphic or Hodgkin's lymphoma)
- EBV status of histology (positive, negative or unknown)
- Site of disease
- Ann Arbor staging at diagnosis (I-IV)

**Immunosuppression:**

- Immunosuppressive agents at the time of diagnosis
- Whether RI was implemented following PTLD diagnosis

**Management and outcomes:**

- Treatment regimens
- Treatment response (complete remission, partial remission, progressive disease)
- Graft survival at last follow up
- Patient survival at last follow up

Cases with missing data were noted and their frequency reported. To facilitate binary data analysis, clinical presentations were categorised into symptom groups. Categories included B symptoms, lymphadenopathy, abdominal symptoms, ENT/oral symptoms, neurological symptoms, respiratory symptoms, urinary symptoms, bleeding/anaemia and incidental findings. Similarly, anatomical sites of disease were categorised into binary variables including involvement in lymph nodes, GIT, liver, spleen, BM, CNS, oral cavity, lung, kidney, bladder and skin/soft tissue. The time of diagnosis of PTLD was defined by the date of biopsy confirming histopathological diagnosis. The date of first presentation proved challenging to accurately determine due to missing, unclear or conflicting documentation across some cases. Where possible, date of initial presentation was defined as the earliest documented clinical encounter with signs and symptoms of PTLD. To enable analysis, categorisation of time from presentation to diagnosis was carried out into 0–2 weeks, 2–4 weeks, 4–8 weeks, 8–12 weeks, and over 12 weeks. PTLD histological classification was recorded according to the WHO 2008 criteria, as this reflects the terminology used in historical pathology reports across the study period.

Statistical analyses were carried out using SPSS software (v30). Categorical data were expressed as percentages while continuous data were expressed as either mean (standard deviation) for normally distributed data or median (interquartile range) for skewed data. Normality was assessed using the Shapiro–Wilk test. Comparisons of normally distributed continuous variables were performed using the independent samples *t*-test, while non-normally distributed continuous variables were compared using the Mann–Whitney *U*-test. Categorical variables were compared using Fisher's exact test. Time-to-event analyses were performed using Kaplan–Meier methods, with differences between groups assessed using the log-rank test. Five-year overall survival was used as the primary survival endpoint. A *p*-value of <0.05 was considered statistically significant.

## 3 Results

### 3.1 Patient demographics

A total of 73 patients diagnosed with PTLD over 22 years from July 2002 to January 2025 met the inclusion criteria and were involved in the analysis. The mean age at PTLD diagnosis was 48.25 years [standard deviation (SD): 13.47] with a median follow up duration of 70 months [interquartile range (IQR): 16–112 months]. Of these patients, 11% (*n* = 8) were aged >65 years and one patient was aged <18 years at diagnosis. The cohort comprised 68% males (*n* = 50) and 32% (*n* = 23) females. Demographics of the cohort are summarised in Table 1.

**Table 1:** Demographics of the PTLD cohort.

Characteristic	All Cases *n* (%)	Early onset < 1 year (%)	Late onset > 1 year (%)	*P*-value
*n*	73	12 (16)	61 (84)	–
Sex				0.564
Male	50 (60)	8 (67)	42 (69)	–
Female	23 (40)	4 (33)	19 (26)	–
Mean age	48.25 (SD: 13.47)	43.75 (SD: 18.111)	49.13 (SD: 12.359)	0.208
Transplant organ				–
Kidney	63 (86)	9 (75)	54 (89)	–
SPK	9 (12)	2 (17)	6 (10)	–
Pancreas-only	1 (1)	0	1 (2)	–
PTLD WHO class				0.362
Monomorphic	51/71 (72)	7/11 (64)	44/60 (73)	–
Polymorphic	12/71 (17)	4/11 (36)	8/60 (13)	–
Early	4/71 (6)	0	4/60 (7)	–
Hodgkin's	4/71 (6)	0	4/60 (7)	–
EBV status				0.007
Positive	30/53 (57)	8/8 (100)	22/45 (49)	–
Negative	23/53 (43)	0	23/45 (51)	–
Ann Arbor stage				0.295
I	10/49 (20)	1/8 (13)	9/41 (22)	–
II	2/49 (4)	1/8 (13)	1/41 (2)	–
III	7/49 (14)	2/8 (25)	5/41 (12)	–
IV	30/49 (61)	4/8 (50)	26/41 (63)	–
Disease site at presentation				0.567
Nodal	48 (66)	8 (67)	40 (66)	–
Extranodal	55 (75)	9 (75)	46 (75)	–
Extranodal sites				–
GIT	24 (33)	6 (50)	18 (30)	–
Liver	7 (10)	1 (8)	6 (10)	–
Spleen	8 (11)	2 (17)	6 (10)	–
BM	4 (6)	1 (8)	3 (5)	–
CNS	4 (6)	1 (8)	3 (5)	–
Oral	6 (8)	0	6 (10)	–
Lung	5 (7)	1 (8)	4 (7)	–
Kidney	5 (7)	1 (8)	4 (7)	–
Bladder	2 (3)	0	2 (3)	–
Adrenal gland	2 (3)	1 (8)	1 (2)	–
Skin/soft tissue	7 (10)	1 (8)	6 (10)	–
Primary site not localised	3 (4)	1 (8)	2 (3)	–
IS at diagnosis				0.261
Single	13/72 (18)	0	13/60 (22)	–
Double	39/72 (54)	7 (58)	32/60 (53)	–
Triple	20/72 (28)	5 (42)	15/60 (25)	–

BM, bone marrow; CNS, central nervous system; EBV, Epstein-Barr virus; GIT, gastrointestinal tract; IS, immunosuppression; PTLD, post-transplant lymphoproliferative disorder; SPK, simultaneous kidney-pancreas; SD, standard deviation; WHO, world health organisation.

The median interval from transplantation to PTLD diagnosis was 132 months (IQR: 60–186). Early onset PTLD (diagnosed within 1 year post-transplant) occurred in 16% (*n* = 12), while late onset PTLD (diagnosed after 1 year) was observed in 84% (*n* = 61). Very late onset PTLD (diagnosed >10 years post-transplant) accounted for 58% (*n* = 41) of cases with 25% (*n* = 18) diagnosed over 15 years and 6% (*n* = 4) beyond the 20th post-transplantation year. Median time from transplant to PTLD diagnosis in early onset disease was 7 months (IQR: 5.5–8.75) and in late onset disease was 146 months (IQR: 96–204).

The majority of patients (86%, *n* = 63) had received renal transplants. In addition, 12% (*n* = 9) of patients had received SPK transplants and one patient had received a pancreas-only transplant. Notably, eight renal transplant recipients and one SPK transplant recipient had previously received renal transplants.

Immunosuppression regimens at PTLD diagnosis involved the use of the calcineurin inhibitors tacrolimus (78%) and cyclosporine (18%), antimetabolites azathioprine (26%) and mycophenolate mofetil (MMF) (45%), mTOR inhibitor sirolimus (3%) and prednisolone (37%). The use of double immunosuppression by tacrolimus and MMF is the standard immunosuppressive regimen at our department following renal transplantation. As such, this combination was most commonly used (32%) followed by triple immunosuppression with tacrolimus, prednisolone and azathioprine (16%).

### 3.2 Clinical presentation

**Figure 1:**
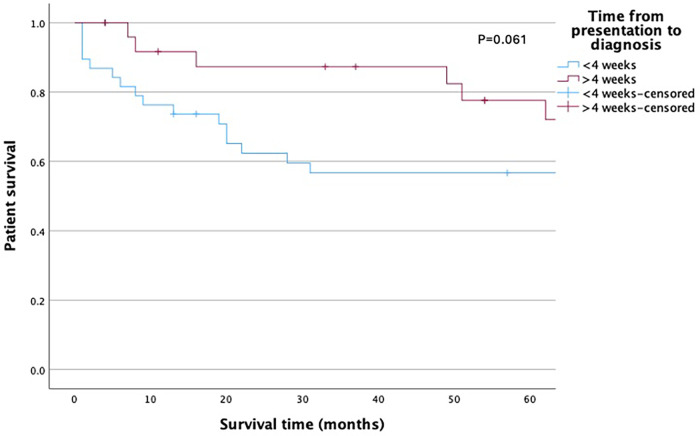
5-year Kaplan–Meier overall survival curves comparing early (<4 weeks) versus delayed (>4 weeks) diagnosis of PTLD.

PTLD demonstrated a heterogenous clinical presentation with abdominal symptoms being the most common. PTLD was identified incidentally in two patients and documentation of presenting symptoms was unavailable in four cases (5%). The median time from first presentation to diagnosis was 28 days although this was unavailable in 14% (*n* = 9) of patients due to incomplete documentation. Patients with bleeding/anaemia as a presenting feature had a significantly prolonged time from presentation to diagnosis (*p* = 0.045). Other symptom categories showed no significant differences in time from presentation to PTLD. Time from presentation to diagnosis of greater than a month had a trend toward reduced survival rates in our cohort (*p* = 0.061) but had no association with graft survival (*p* = 0.787). Kaplan–Meier survival curves comparing overall survival in patients with and without a diagnostic delay (>4 weeks) are demonstrated in Figure 1. A breakdown of symptom categories and their time from presentation to diagnosis is displayed in Table 2.

**Table 2:** Presenting features of the PTLD cohort according to time from presentation to diagnosis.

Symptom category	Time from presentation to diagnosis, weeks							
	All cases, n	0–2	2–4	4–8	8–12	>12	Unknown	Median time to diagnosis days (IQR)
Abdominal	28	12	8	6	2	0	0	24.5 (9.25–40)
B symptoms	27	6	6	3	5	3	3	29 (11.25–77.5)
Lymphadenopathy	12	3	2	0	3	2	2	23 (4–134.5)
ENT/oral	9	2	0	1	2	2	2	41 (5–89)
Bleeding/anaemia	11	2	2	2	2	3	0	60 (21–127)
Neurological	3	2	0	0	1	0	0	9 (range: 6–86)
Respiratory	8	2	3	1	0	1	0	16 (5–28.25)
Urinary	5	0	3	0	0	2	0	30 (22–124.25)
Incidental	2	1	0	0	0	1	0	

IQR, Interquartile range.

#### 3.2.1 Abdominal symptoms

Abdominal symptoms were the most frequent presenting feature occurring in 41% (*n* = 28) of patients. These included abdominal pain, bowel obstruction, bowel perforation, reflux, distention, change in bowel habit, GI bleeding and reduced appetite. Three patients presented with bowel perforation requiring urgent surgical resection. Among those with abdominal symptoms, 32% (*n* = 9) also reported B symptoms, 18% (*n* = 5) bleeding/anaemia and 4% (*n* = 1) urinary symptoms. The most frequent anatomical sites of disease in this subgroup were lymph nodes (68%, *n* = 19) and GIT (64%, *n* = 18). Additionally, there was involvement of the liver (11%, *n* = 3), the spleen, BM, lungs and intraabdominal soft tissue (each 7%, *n* = 2). The median time from transplant to PTLD diagnosis in these patients was 137 months (IQR: 86.25–167.25).

Diagnostic biopsies in this group were primarily surgical (39%, *n* = 11), including both laparotomy and laparoscopy, and endoscopic techniques (32%, *n* = 9) comprising colonoscopy (*n* = 4) and OGD (*n* = 5). Complete remission or partial remission occurred in 7% (*n* = 19) while progressive disease was observed in 28% (*n* = 7). One patient had ongoing treatment with their outcome yet to be determined.

#### 3.2.2 B symptoms

Systemic B symptoms were present in 39% (*n* = 27) of patients at presentation. Coexisting symptoms were varied in nature and included lymphadenopathy (19%, *n* = 5), bleeding/anaemia (11%, *n* = 3) and respiratory symptoms (11%, *n* = 3). Urinary, respiratory, neurological and ENT/oral symptoms were also seen in one patient each. Anatomical distribution of disease in this subgroup revealed high rates of lymph node involvement (85%, *n* = 23), followed by involvement of the spleen (22%, *n* = 6) and GIT (19%, *n* = 5). The median time from transplant to PTLD in these patients was 108 months (IQR: 35–165). In terms of outcomes, 59% (*n* = 16) of patients with B symptoms achieved complete remission, 15% (*n* = 4) had partial remission and 22% (*n* = 6) had progressive disease. One patient remained on active treatment.

#### 3.2.3 Neurological symptoms

Neurological symptoms were identified in three (4%) patients. The first patient experienced seizures and left sided weakness, the second acute confusion and third tremors and memory loss as well as decreased appetite and weight loss. The first two patients were on triple IS and the third was on single IS. All were monomorphic cases affecting the CNS, with the first two EBV-positive and third unknown, diagnosed by brain biopsy. The first patient received four rounds of rituximab along with ibrutinib. The second was enrolled onto the TIDAL trial and subsequently given four rounds of rituximab and ibrutinib. The third patient received 20 cycles of radiotherapy to the brain. The first patient had progressive disease and subsequent death within a year, the second had complete remission and the third had ongoing treatment. The first two patients retained graft function while the third experienced graft failure prior to PTLD diagnosis.

#### 3.2.4 Other presenting features

Lymphadenopathy was reported in 16% (*n* = 11) of patients. All had nodal involvement, with 45% also demonstrating extranodal spread (*n* = 5), including to the BM (*n* = 2), GIT, liver, spleen, CNS, oral cavity, lung, kidney, adrenal gland and skin/soft tissue (each *n* = 1). Diagnoses were made primarily by lymph node biopsy (82%, *n* = 9). The majority of these patients achieved complete remission (91%, *n* = 10) and one experienced progressive disease. The 3-year survival rate was 100% in this subgroup. Graft loss occurred in around half of patients (45%, *n* = 5).

ENT and oral cavity-related symptoms were reported in 13% (*n* = 9) patients constituting symptoms of sore throat, dysphagia, tonsillar mass, oral ulcers and dental discomfort. Occurring alongside were respiratory symptoms (*n* = 3) and bleeding/anaemia (*n* = 1). Disease localisation in this group was primarily nodal (67%, *n* = 6) and in the oral cavity (56%, *n* = 5) with some involvement in the GIT, liver, spleen, BM, lung, kidney and adrenal gland (each *n* = 1). Most common diagnostic modality was by soft tissue biopsy (44%, *n* = 4). Complete remission was achieved in 89% (*n* = 8) of patients. Three-year survival in this group was 100% with one case of graft loss.

Presenting features of bleeding and/or anaemia occurred in 16% (*n* = 11), with manifestations including anaemia (*n* = 7), lower GI bleeding (*n* = 2) and melaena (*n* = 2). Near half (45%, *n* = 5) also presented with additional abdominal symptoms. Diagnostic modalities varied but primarily included endoscopy (64%, *n* = 7), followed by organ biopsy (*n* = 2) and surgical biopsy (*n* = 2). Median interval from symptom onset to PTLD diagnosis in this group was significantly longer at 60 days (IQR: 21–127, *p* = 0.045) compared to other subgroups. Three-year patient survival was 82% (*n* = 9) and graft loss occurred in two patients.

Respiratory symptoms were noted in 12% (*n* = 8) of patients with the most commonly reported being shortness of breath and chest pain. Lymph node involvement was seen in 88% (*n* = 7) with pulmonary or oral cavity involvement in 25% (*n* = 2). Median time from transplant to PTLD in this group was 162 months (IQR: 38.25–225.75). Three-year survival was calculated as 75%. 88% (*n* = 7) achieved complete remission or partial remission. Seven patients retained graft function with the graft status of one patient unknown.

Urinary symptoms consisting of dysuria, haematuria, oliguria and urinary frequency were least common, seen in 9% (*n* = 6) of patients. Sites of disease involved lymph nodes (*n* = 4), kidney (*n* = 3), bladder (*n* = 2), skin/soft tissue (*n* = 2) and lungs (*n* = 1). Half achieved complete remission while the other half experienced progressive disease. Three-year survival was 50% and all patients retained graft function following PTLD.

### 3.3 Disease classification and diagnostic modality

Classification according to the 2008 WHO criteria is summarised in Table 1 and was available in 71 patients. Monomorphic PTLD was the most prevalent subtype occurring in 72% (*n* = 51) of patients with diffuse large B cell lymphoma (DLBCL) being the predominant histology (*n* = 33). Information on EBV status of PTLD tissue was available for 73% (*n* = 53) of patients. PTLD histology was EBV-positive in 30 cases (57%). All early onset PTLD cases (*n* = 8) were EBV-positive. Median time from transplant to diagnosis in EBV-positive cases was 95 months (IQR: 9–195) and 156 months (IQR: 108–180) for EBV-negative cases. Ann Arbor staging was determined for 67% (*n* = 49) patients at diagnosis. Of these, 76% (*n* = 37) were at stage 3 or 4.

Diagnostic modalities were identified in 88% (*n* = 64) of patients. The main method of histological diagnosis was by lymph node biopsy carried out in 27% (*n* = 17) of patients, followed by endoscopic biopsy carried out in 25% (*n* = 16). Endoscopy consisted of colonoscopy (*n* = 7), oesophagogastroduodenoscopy (OGD) (*n* = 6), bronchoscopy (*n* = 2) and cystoscopy (*n* = 1). Surgical biopsy was carried out on 22% (*n* = 14) of patients, needle biopsy in 11% (*n* = 7) and soft tissue biopsy in 8% (*n* = 5). Additionally, bone marrow biopsies were the diagnostic method of choice in two patients and brain biopsy in three.

### 3.4 Treatment

Treatment for PTLD at our centre typically involves systemic therapy spanning approximately 18 weeks, comprising immunochemotherapy and/or targeted therapies. During active treatment, EBV polymerase chain reaction (PCR) is monitored closely at regular intervals (typically every 2–3 weeks). Following the cessation of treatment, surveillance EBV PCR testing is carried out every 3–4 months for the first 2 years, and every 6 months for an additional year. In cases of a rising EBV viral load during the post-treatment follow-up period, further radiological investigation with CT or PET-CT is promptly undertaken to evaluate for disease relapse, with subsequent management tailored accordingly.

Within our cohort, treatment protocols included RI in 93% of patients. The primary reasons for maintaining immunosuppression included high risk of graft failure and multiple comorbidities leading to palliation. Initial rituximab monotherapy was administered in 27% (*n* = 20) of cases, RCHOP chemotherapy in 52% (*n* = 38) of cases and intrathecal (IT) or intravenous (IV) methotrexate in 18% (*n* = 13). Surgical procedures, primarily used for emergency treatment or diagnostic purposes, were carried out in 23% (*n* = 17) of patients. Nine patients received rituximab monotherapy followed by CHOP chemotherapy. Surgical procedures included bowel resection (*n* = 11), tonsillectomy (*n* = 3), splenectomy (*n* = 2) and right hemicolectomy with stent ileostomy (*n* = 1). Other less common forms of treatment included radiotherapy in (8%, *n* = 6), stem cell therapy in (4%, *n* = 3), other forms of chemotherapy (18%, *n* = 13) and palliative or conservative management (7%, *n* = 5). Additionally, three patients received ibrutinib and two received anti-EBV cytotoxic T lymphocytes (CTLs).

RI alone was used as treatment for 42% (*n* = 5) of patients with polymorphic PTLD compared to 2% (*n* = 1) in monomorphic PTLD. Initial rituximab monotherapy was used in 42% (*n* = 5) of polymorphic patients and 27% (*n* = 14) of monomorphic patients. RCHOP was administered in only one (8%) polymorphic patient and 71% (*n* = 36) of monomorphic patients. All four patients with Hodgkin's lymphoma received two to six cycles of ABVD chemotherapy each and all achieved complete remission.

### 3.5 Outcomes

**Figure 2:**
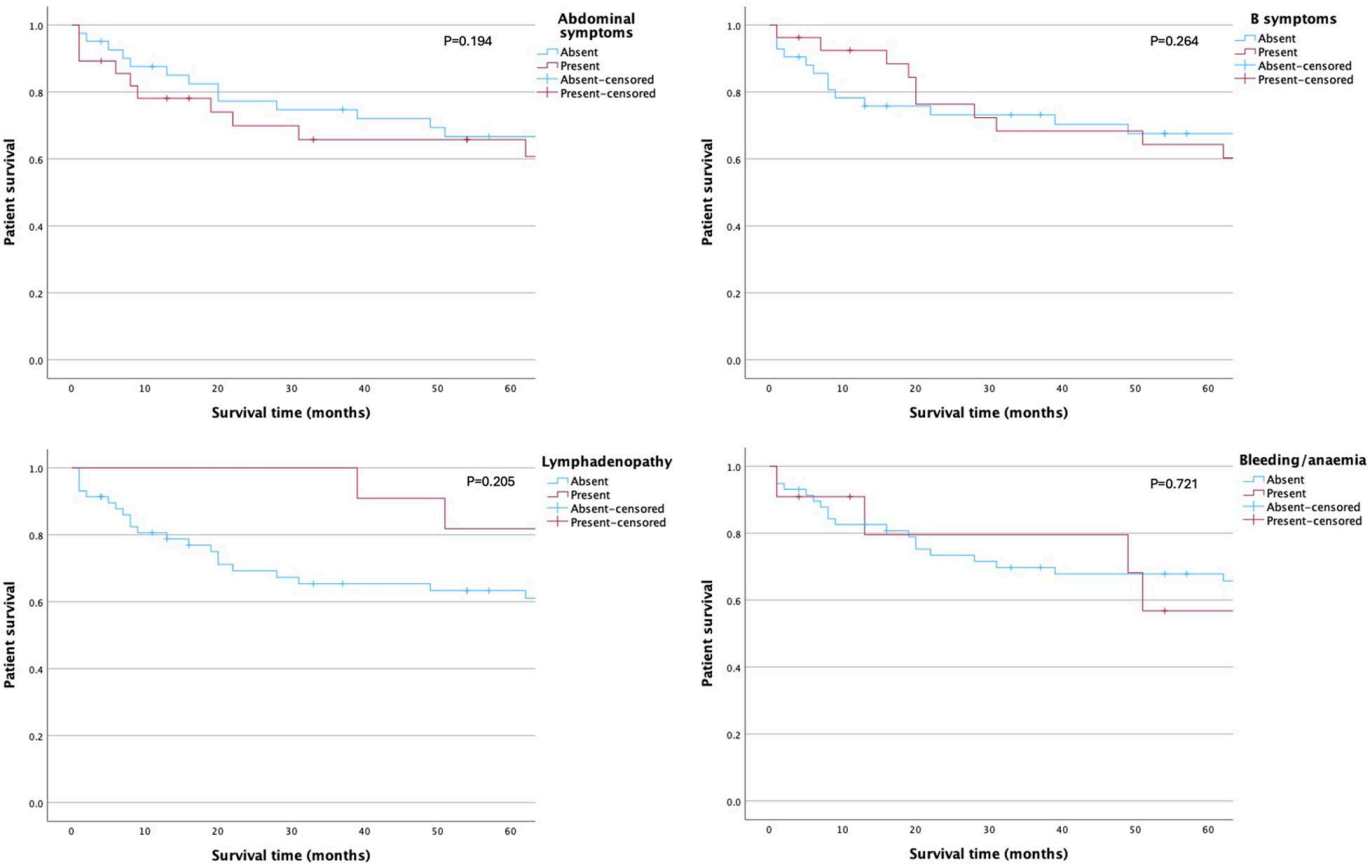
5-year Kaplan–Meier overall survival curves for different symptom categories.

Mortality rates were 17% (*n* = 12) at 1 year, 27% (*n* = 20) at 3 years and 32% (*n* = 23) at 5 years post-PTLD diagnosis. During the follow up period, a total of 35 patients (48%) died. Median overall survival was not reached during the follow-up period. Among the 35 patients who died, the median time from PTLD diagnosis to death was 22 months (IQR: 7–72). Complete remission, defined as the clinical and/or radiological resolution of disease, was achieved in 68% (*n* = 50) of patients including seven who responded to RI alone. Partial remission was observed in 6% (*n* = 4) and progressive disease in 22% (*n* = 16). Three patients had ongoing treatment with their outcomes yet to be determined. Treatment response (complete or partial remission) was seen in 93% of EBV-positive cases and 71% of EBV-negative cases. A breakdown of treatment outcomes and their time from presentation to diagnosis is displayed in Table 3. Death-censored graft loss was observed in 18% (*n* = 13) of patients involving 12 kidney grafts and one pancreas graft. The median time from PTLD to graft loss was 16 months (IQR: 9–53.5). Four patients experienced graft failure prior to PTLD diagnosis—two with kidney failure and two SPK-recipients with pancreas failure. Kaplan–Meier survival curves stratified by common presenting symptoms are shown in Figure 2.

**Table 3:** Treatment outcomes of the PTLD cohort according to time from presentation to diagnosis.

Symptom category	All cases, n	Time from presentation to diagnosis, weeks				
		0–2	2–4	4–8	8–12	>12
CR	41	15	10	6	4	6
PR	4	0	0	2	0	2
PD	15	5	6	0	3	1
Ongoing treatment	2	1	0	0	0	1

CR, Complete remission; PD, Progressive disease; PR, Partial remission.

## 4 Discussion

This retrospective evaluation provides a comprehensive analysis of the clinical presentations, diagnostic approaches, treatment protocols and outcomes in a cohort of 73 PTLD patients at a large tertiary transplant centre over a 22-year period. Our findings reaffirm the diversity of PTLD and identify potential areas for service-level improvement, particularly in regard to early recognition and referral pathways.

### 4.1 Disease characteristics

Monomorphic PTLD, particularly DLBCL, represented the dominant histological subtype. Polymorphic and early lesions were less prevalent and were often treated with RI alone due to their more indolent clinical course, in line with current BHS guidelines (24). EBV status of histology was a near-equal split between positive (57%) and negative (43%) cases. However, EBV status of PTLD tissue was unavailable in 27% of cases, limiting full disease classification. The BSH guidelines on the management of PTLD in SOT recipients recommends the inclusion of EBV-encoded RNA *in situ* hybridisation (EBER ISH) on biopsy specimens in the diagnostic work up for PTLD (24, 25). Missing EBV status data in our cohort likely reflects historic practices predating implementation of guidelines and limitations in available tissue. Nonetheless, mandating EBER ISH testing on PTLD specimens would allow for improved therapeutic decision-making and ensure alignment with national standards. The relationship between EBV and PTLD, particularly in early-onset PTLD, is widely recognised (29). This was demonstrated in our cohort with all cases of early onset PTLD exhibiting EBV positivity. In contrast, late-onset PTLD accounted for 84% of all cases with nearly 60% occurring over 10 years post-transplant. This supports growing evidence that PTLD, particularly EBV-negative disease, is increasingly a late complication (30). Compared to a French registry (31) our incidence of early onset PTLD was lower (16% vs. 48%) reflecting this trend. The poorer prognosis typically seen with EBV-negativity was demonstrated in our cohort given treatment response was lower in comparison to EBV-positive cases (71% vs. 90%).

### 4.2 Diagnostic and clinical implications

The median time from transplant to PTLD diagnosis was 132 months (11 years), notably longer than prior studies reporting median intervals of 48–104 months (5, 11, 31–33), although many of these studies analysed multi-organ transplant cohorts. The prevalence of PTLD onset >10 years after transplantation reinforces the need for long-term surveillance well beyond the conventional 5–10 year post-transplant window in renal transplant recipients especially as late onset PTLD is associated with poorer prognosis (34). The aetiology of late onset disease is complex and postulated to be the result of improved graft survival leading to longer periods of immunosuppression. Consequently, PTLD continues to be a long-term risk in our population.

Incidence of PTLD in our cohort demonstrated a bimodal distribution: an early peak within a year of transplant and a broader peak 10 years post-transplant, consistent with published patterns (5, 12, 17). Of note, four patients in our cohort developed PTLD despite established graft failure, contrary to prior studies suggesting minimal PTLD risk in patients with graft loss (5). This challenges current assumptions and suggests that PTLD is not limited to patients with stable graft function on immunosuppression and should remain a differential in patients with failed grafts.

Clinical presentations were varied with abdominal symptoms and B symptoms being the most common. Our data demonstrated moderate overlap between clinical presentations which can complicate clinical assessment. Given the predominance of abdominal symptoms in our cohort, a low index of clinical suspicion would be beneficial for non-specific GI symptoms. Notably, three patients presented with bowel perforation, all of whom required surgical resection, demonstrating the ability of PTLD to present acutely and mimic surgical emergencies. Presentations involving bleeding and/or anaemia were associated with significant diagnostic delays. However, anaemia in PTLD may be multifactorial and not necessarily attributable to blood loss. Symptom overlap and heterogeneity within this group limit definitive conclusions. The introduction of an alert system for persistent anaemia or unexplained GI bleeding in transplant recipients at our centre may help facilitate earlier investigation of PTLD.

Delays in diagnosis of over a month had near significant reduction in overall survival. Nevertheless, a median time to definitive diagnosis of 28 days highlights potential delays, particularly in larger referral networks. Reducing time to diagnosis via lower clinical thresholds for biopsy may improve outcomes. A quality improvement initiative looking into the number of referrals made for PTLD patients treated at our centre could identify bottlenecks contributing to these delays.

### 4.3 Anatomical sites of disease

Extranodal disease was prominent in our cohort (75%), conforming to what is typical of PTLD. Anatomical sites of disease were varied with 33% occurring in the GIT making it the most common site of PTLD in our cohort, consistent with other series on renal transplant recipients (4, 20, 35). This further consolidates the need for a low threshold for abdominal imaging and endoscopy in transplant recipients presenting with abdominal symptoms.

### 4.4 Therapeutic strategies and outcomes

Given the heterogeneity in histology and staging of disease, the development of a standardised treatment approach for PTLD remains challenging. This was evident in our cohort where a wide variety of treatment approaches was observed. RI was near-universal (93%) remaining first line in PTLD management consistent with current BSH guidelines (24). At our centre, RI is typically individualised, often involving stepwise withdrawal of agents such as antiproliferative therapies (typically MMF), followed by adjustment of calcineurin inhibitors where appropriate; however, approaches vary depending on patient factors, graft function and disease severity. Since its inception in the use of PTLD, rituximab has been shown to be a highly efficacious form of treatment (27, 36). Its use has become the standard of care in our cohort and was widely seen as patients receiving rituximab either as monotherapy or in combination with chemotherapy.

Recent years have seen a shift in the management of relapsed or refractory PTLD, particularly in cases unresponsive to chemotherapy regimens such as RCHOP. The emergence of cellular immunotherapies, such as anti-EBV CTLs, has demonstrated promising efficacy in the management of PTLD, particularly in EBV-positive disease (37). In our cohort, two patients received anti-EBV CTLs and both achieved complete remission. As manufacturing processes and accessibility improve, the broader application of these therapies may be anticipated. Additionally, the use of chimeric antigen receptor (CAR) T-cell therapy has demonstrated encouraging results in early studies involving PTLD (38–40). However, its application in transplant recipients presents unique challenges. The manufacturing process requires time, during which disease progression may occur, and typically necessitates reduction or temporary discontinuation of immunosuppressive therapy. This introduces a significant risk of graft rejection, particularly in solid organ transplant recipients. Although CAR T-cell therapy is not yet established in our centre's clinical practice, one patient outside of the cohort study has recently commenced treatment. Ongoing large-scale studies are required to better define its long-term efficacy, safety and feasibility within the context of transplant-specific immunosuppression.

The 1-year mortality in our PTLD cohort was 17%; this was lower than the 40% reported by Opelz and Döhler (4), 52% by Cheung et al. (32) and 27% by the French registry (31). Our overall treatment response rate was 62%, also exceeding the 52% reported by Cheung et al. (32). Although many of these papers included patients from the pre-rituximab era which may partly account for the improved outcomes observed in our cohort. When stratified by different symptom groups, survival outcomes in our cohort appeared to differ: patients with lymphadenopathy and ENT/oral symptoms had favourable 3-year survival (89%–100%) while those with CNS or urinary symptoms had poorer prognoses (50%–66%).

At our transplant centre, there is no PTLD registry making identification of patients for quality improvement projects and research difficult. Our department would benefit from establishing a dedicated PTLD registry to enable gaps in care and patterns in PTLD presentations and management be identified and acted upon. The establishment of national or multicentre registries is essential for refining risk stratification in this population.

### 4.5 Strengths and limitations

This study benefits from the use of a large cohort with a long follow up period (22 years) allowing the capture of late-onset PTLD cases occurring over 15–20 years post-transplant. The MRI renal transplant unit is a high-volume tertiary centre serving North West England allowing collection of data from a large, diverse population and identification of local patterns.

The study is limited by its retrospective nature and missing data in key variables, however there was no evidence to suggest bias in missing data. Estimations of first presentation and diagnostic delays for some cases were reliant on electronic documentation and may have been affected by missing or ambiguous records, particularly from cases occurring early in the cohort. The sample size, although large for a PTLD cohort, limits the generalisability of the findings.

## 5 Conclusion

In summary, PTLD remains a clinically significant and complex complication in our renal transplant recipients. The variability in clinical presentations and the increasing prevalence of late-onset EBV-negative PTLD highlights the need for long-term vigilance. Future initiatives should focus on improving clinical awareness of late-onset PTLD, streamlining referral pathways to reduce diagnostic delay, engaging with research on emerging immunotherapies and establishing a national PTLD registry to support further studies.

## Data Availability

The raw data supporting the conclusions of this article will be made available by the authors, without undue reservation.
